# Impact of COVID-19 social-distancing on sleep timing and duration during a university semester

**DOI:** 10.1371/journal.pone.0250793

**Published:** 2021-04-26

**Authors:** Andrea N. Smit, Myriam Juda, Ashley Livingstone, Stephanie R. U., Ralph E. Mistlberger

**Affiliations:** 1 Department of Psychology, Simon Fraser University, Burnaby, Canada; 2 Department of Psychiatry, Now at the BRAIN Lab, Institute of Mental Health, University of British Columbia, Vancouver, Canada; University of Lübeck, GERMANY

## Abstract

Social-distancing directives to contain community transmission of the COVID-19 virus can be expected to affect sleep timing, duration or quality. Remote work or school may increase time available for sleep, with benefits for immune function and mental health, particularly in those individuals who obtain less sleep than age-adjusted recommendations. Young adults are thought to regularly carry significant sleep debt related in part to misalignment between endogenous circadian clock time and social time. We examined the impact of social-distancing measures on sleep in young adults by comparing sleep self-studies submitted by students enrolled in a university course during the 2020 summer session (entirely remote instruction, N = 80) with self-studies submitted by students enrolled in the same course during previous summer semesters (on-campus instruction, N = 452; cross-sectional study design). Self-studies included 2–8 week sleep diaries, two chronotype questionnaires, written reports, and sleep tracker (Fitbit) data from a subsample. Students in the 2020 remote instruction semester slept later, less efficiently, less at night and more in the day, but did not sleep more overall despite online, asynchronous classes and ~44% fewer work days compared to students in previous summers. Subjectively, the net impact on sleep was judged as positive or negative in equal numbers of students, with students identifying as evening types significantly more likely to report a positive impact, and morning types a negative impact. Several features of the data suggest that the average amount of sleep reported by students in this summer course, historically and during the 2020 remote school semester, represents a homeostatic balance, rather than a chronic deficit. Regardless of the interpretation, the results provide additional evidence that social-distancing measures affect sleep in heterogeneous ways.

## 1. Introduction

There is a widely held, albeit not unanimous, perspective that a substantial portion of the population in the industrialized world regularly experiences insufficient sleep, due to competing time demands from work, school and social life [1–8]. This may be particularly acute in young adults, a demographic that exhibits greater misalignment between circadian clock time and social time (i.e., social jetlag), due to an age-related tendency toward a delayed phase of circadian entrainment (evening chronotype) [9,10]. One consequence of social jetlag is that on work days, the social clock (work or school) interrupts sleep before completion of the biological night. Increased sleep on subsequent free days (typically weekends) is interpreted as evidence that the amount of sleep on work days does not fully meet physiological requirements, and must be compensated on free days to maintain homeostatic balance [10]. Sleep homeostasis is a non-linear process, achieved in part by changes in sleep intensity [11]. Consequently, every hour of sleep restriction on work days does not need to be repaid by an extra hour of sleep on free days. If social jetlag does cause significant physiological sleep restriction, and if recovery from sleep restriction is non-linear, then people with a mix of work and free days who experience social jetlag should over time average less sleep per day than they would without work days. With sufficient sample sizes, it should be possible to see this in cross-sectional data, comparing age- and sex-matched groups with different proportions of work days and degrees of social jetlag.

During the Coronavirus 2019 (COVID-19) pandemic, social-distancing (variously labelled social-restriction, shelter-in-place, stay-at-home, lockdown, circuit breaker, quarantine, curfew) was a central component of the public health measures taken to contain community transmission. In British Columbia, Canada, as in many jurisdictions elsewhere, this included a transition to remote schooling, and precipitated a contraction in many work sectors that typically employ young adults. With no need to commute to campus, fewer off-campus work opportunities, and public health recommendations to stay at home, constraints on sleep timing and duration for many individuals would be relaxed. This provided a unique opportunity to examine the extent to which sleep may normally be in deficit in young adult university students. If university students are habitually sleep restricted during a typical university semester, and more time is made available for sleep, then average daily sleep duration should increase by some detectable amount, at night or through increased daytime napping.

Evidence available to date from on-line survey studies suggests that the effects of COVID-19 social-distancing on sleep are heterogeneous, and likely vary with the study population. Widely reported effects include later bedtimes, increased time-in-bed (TIB, a proxy for sleep), decreased subjective sleep quality and increased prevalence of insomnia or other sleep disruptions, e.g., [12–19]. Some studies have reported shorter nocturnal TIB independent of insomnia, no change in TIB, or improved sleep quality [15–21]. Studies accessing large scale databases from wearable [22] or smartphone crowdsourcing technology [23,24] report that at the population level, sleep was ~20 minutes later and ~20 minutes longer during social-distancing. Only one study to our knowledge collected TIB data prospectively from young adult university students, using a daily log to record bedtime and wakeup time for a week prior to and again during public ’Stay-At-Home’ orders [25]. Compared to the baseline week (late January, 2020), TIB during the stay-at-home week (late April, 2020), was later by ~32 min and longer by ~26 min (averaging weekdays and weekends).

We were able to examine the impact of social-distancing measures on sleep in young adults by comparing sleep self-studies submitted by students enrolled in a university course during the 2020 summer session (remote instruction) with self-studies submitted by students enrolled in the same course during previous summer semesters (on-campus instruction). The results are generally consistent with findings from studies of other populations, although we do not see an increase in sleep duration in the 2020 group compared to previous years, despite a marked reduction in self-classified work days.

## 2. Materials and methods

### 2.1. Participants

Participants were students enrolled in an upper division undergraduate biopsychology course on sleep and biological rhythms at Simon Fraser University (Vancouver, BC, Canada; 49.3^o^N, 122.9^o^W). Data are presented here from 452 students (71% female, 23.8 ± 4.7 y) enrolled during summer semesters from 2009–2013 (hereafter, the ’archival’ sample) and from 80 students (72% female, 22.7 ± 4.7 y) enrolled during the summer semester of 2020, when all courses were delivered remotely due to COVID-19 social-distancing measures. A minimal risk study protocol was approved by the institutional research ethics board at Simon Fraser University (#2020s0392).

### 2.2. Measures and procedure

All students conducted a sleep self-study for partial course credit. Elements of the self-study included a structured sleep diary (minimum 14 consecutive days), the Morningness-Eveningness Questionnaire (MEQ) [26], and the Munich Chronotype Questionnaire (MCTQ) [27]. All students submitted diaries. MEQ and MCTQ data were obtained from 441 students in the archive group, and 79 students in the 2020 group. For the 2020 semester, students were required to also use a sleep tracker or smartphone app of their choosing (free or for purchase) for comparison with diary data, and to submit a report that included a discussion of how social distancing measures, particularly remote work and school, might impact sleep generally and their own sleep specifically. The questionnaires were completed via an online web survey tool during the first few weeks of the semester (typically mid-to-late May). The diaries and sleep tracker data were collected during the middle of the semester, typically June to late July, and reports submitted at the end of July.

The MEQ asks questions about preferred and ’feeling best’ times for sleep and waking activities, and yields a score ranging from 16–86, where a higher score indicates a greater morning preference. The MCTQ asks for typical bedtime, sleep onset time, wakeup time, frequency of alarm use, nap time, nap duration, and time outdoors. Information is entered separately for "work" and "free" days. Free days were defined as days when participants were free to wake at their preferred time. Work days were defined as days when participants were required to wake for social obligations (including school). This might typically involve use of an alarm, but alarm use per se was not used to define work and free days. Sleep diary data were entered daily. Required diary entries included bedtime, estimated sleep-onset-latency (SOL), wake-after-sleep-onset (WASO) if recalled, wake-up time and any daytime naps (time and duration). During the 2020 semester, time of first outdoor daylight exposure and estimated total time outdoors were also required. Optional measures included mealtimes, exercise, and caffeine intake. Students were instructed to record nocturnal sleep information in the morning, and daytime information at bedtime.

### 2.3. Sleep variables

Sleep onset and wakeup times were used to calculate nocturnal sleep duration and midsleep time, defined as the midpoint between sleep onset and sleep end. Midsleep was calculated separately for work days (MSW) and free days (MSF), and separately for the MCTQ (MSW_MCTQ_; MSF_MCTQ_) and sleep diary (MSW_Diary_; MSF_Diary_). MSF is used as the primary phase reference point to estimate chronotype (phase of entrainment to local time) because it is assumed to more accurately reflect biological time rather than social time. It has been recommended that MSF be corrected for sleep debt that may accumulate on work days [28]. This is based on the reasonable assumption that changes in sleep timing on free-days may be partly due to homeostatic regulation of sleep duration, which introduces error into MSF as a measure of chronotype. The correction assumes that sleep debt accumulates linearly across the work week, and is compensated by a linear increase in sleep duration on free days. Both assumptions are debatable, given that compensation for lost sleep is a non-linear process [11]. Also, by contrast with the conventional ‘5 days on, 2 days off’ work week, university students in Canada typically have irregular school and work schedules. There may be fewer constraints on napping during school days, and these may compensate for sleep debt prior to free days. With these considerations in mind, midsleep was examined without the sleep correction, unless stated otherwise and identified as MSFsc. For some analyses, sleep timing and duration were averaged across all days; midsleep time for all days correlates more strongly with physiological measures of circadian phase (e.g., dim-light-melatonin-onset, DLMO) than does MSF or MSFsc [29].

Differences in sleep timing between work and free days may be due to misalignment between biological (circadian) time and social time. This has been conceptualized as ’social jetlag’, with work and free days representing different social time zones [10]. Social jetlag was quantified as the difference between midsleep on work and free days (SJMS = MSF—MSW). Both signed and absolute values were examined. Misalignment can be expected to affect sleep duration on work days or free days, depending on chronotype. Differences in sleep duration between work and free days were therefore also calculated (SDF—SDW).

## 2.4. Qualitative analyses

Students in the 2020 group were required to submit a structured written report on the results of their sleep self-study. Required discussion topics relevant to the current study included 1. assessment of the correspondence between diary, questionnaire and sleep tracker data, 2. assessment of their own sleep duration and chronotype relative to population norms, 3. factors that influence their sleep, and 4. the impact of COVID-19 social-distancing measures on their sleep. Whether the lifestyle changes forced by social-distancing measures would be experienced as beneficial or detrimental to sleep was an open question. To gauge the students’ experience, the reports were evaluated by the course instructor (R.E.M.) for elements that could be assigned a positive (+1) or negative (-1) value (null value (0) if absent). Positive elements were 1. more consistent bedtimes and wakeup times, 2. more time in bed (unless students explicitly stated that this was not a positive), 3. feeling more rested, 4. better subjective sleep quality, 5. sleep overall satisfactory. Negative elements were 1. less consistent bedtimes and waketimes, 2. less perceived sleep, 3. less rested, 4. problems falling or staying asleep, 5. excessive delay of sleep timing (if perceived as a problem). Other elements that contributed to the assessment included statements concerning insufficient daily sunlight exposure, insufficient exercise, and lack of structure, where these were felt to impact sleep. The assessments were made blind to the sleep timing and duration data for each student.

### 2.5. Data treatment and statistical analyses

For statistical analyses and graphing (Prism 9.0.1, GraphPad Software, San Diego USA), clock times were converted to real numbers. Sleep variables that were normally distributed were compared within- or between-groups using 1-way Analysis of Variance (ANOVA, with Dunn’s multiple comparisons tests), mixed model 2-way ANOVA (with Sidak multiple comparisons tests), or t-tests. Variables that failed normality tests were compared between groups using the Mann-Whitney U test and within groups using Wilcoxon matched pairs signed rank tests. Pearson r correlations were used to quantify relationships between variables. Means in the text are reported with standard deviations.

## 3. Results

### 3.1. Student sleep diaries correspond well with Fitbit and questionnaire data

Participants in sleep studies are typically volunteers. The sleep diary and questionnaire data presented here are from undergraduate student projects assigned for partial course credit. It has been suggested that sleep diaries submitted as a course requirement may not be as reliable as those submitted by volunteers. If so, we would predict a poor correspondence between student sleep diaries and other measures of sleep timing, such as actimetry or questionnaires (retrospective, but requiring little effort to complete, and thus unlikely to be faked).

To address this issue, we compared sleep diary data with automated sleep tracker data from a subset of 19 students in the 2020 summer sample who wore recent model Fitbit wristbands (Inspire HR, Charge HR, Charge 2,3,4). Fitbit estimates of sleep timing and duration show acceptable sensitivity and specificity by comparison with Actiwatches and polysomnography [30,31]. Sleep diary data were also compared with MCTQ estimates of sleep timing and duration, and with the MEQ measure of morning-evening preference. Responses on these questionnaires have been shown to correlate strongly with sleep diary data and with physiological measures of circadian phase, such as dim-light melatonin onset, in volunteer subjects [27,28,32,33].

**Fig 1:**
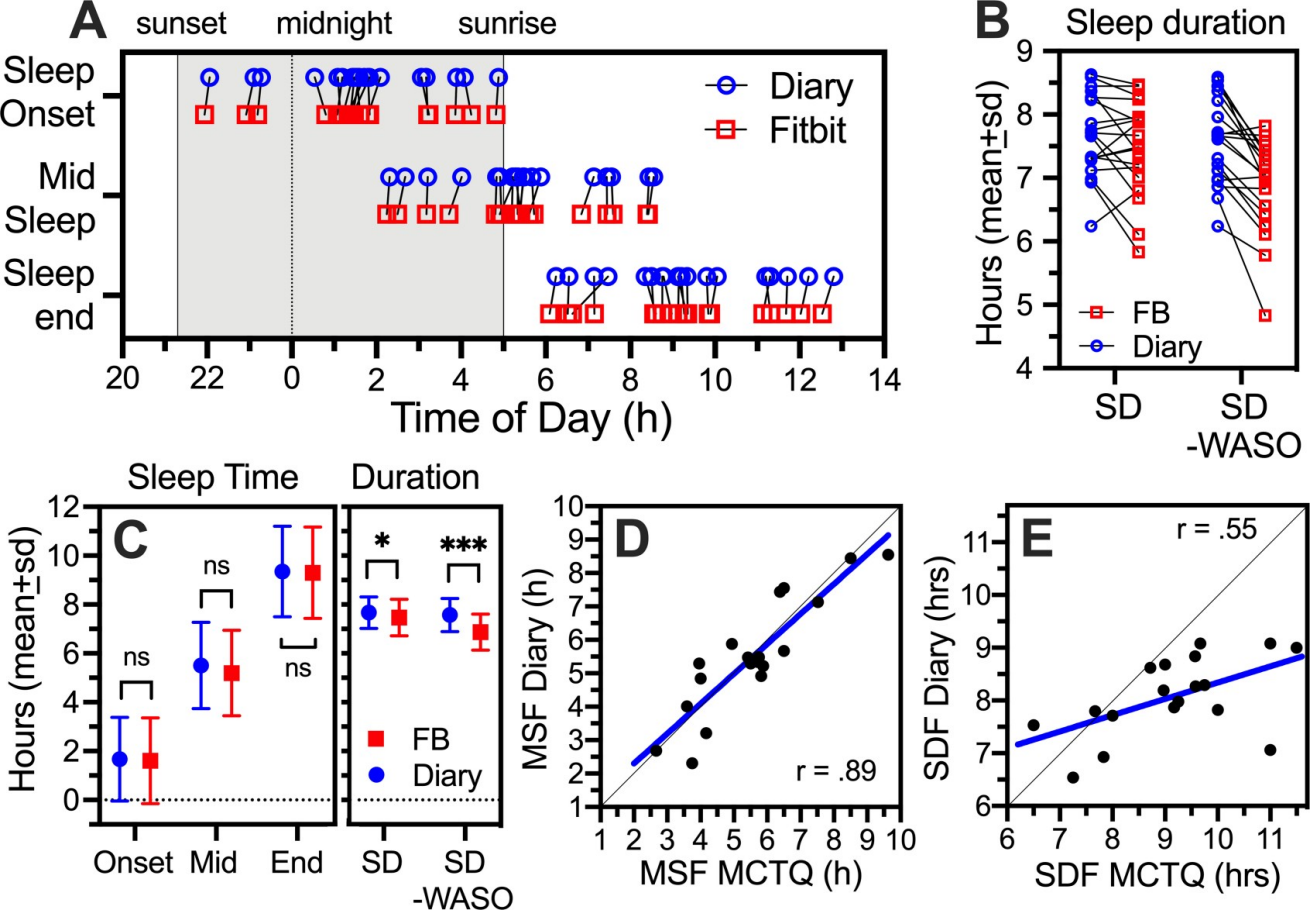
Comparison of sleep timing and duration from sleep diaries and Fitbit (FB) sleep trackers from a 2020 subgroup (N = 19). [A]. Sleep onset, midsleep and sleep end in individual subjects based on sleep diaries (blue circles) and Fitbits (red squares). Grey shading denotes night time, with sunset and sunrise times corresponding to late June in Vancouver, Canada. Hour 0 denotes midnight. [B]. Sleep duration (SD), in hours between sleep onset and end, and adjusted for reported wake-after-sleep-onset (SD-WASO). [C]. Group mean (± standard deviation) sleep onset, midsleep, sleep end, and sleep duration, from diary and FB data. [D]. Scatterplot of midsleep on free days (MSF) from diary and MCTQ data. [E]. Scatterplot of sleep duration on free days (SDF) from diary and MCTQ data. Paired t-tests, *p < .05, ***p < .001.

A plot of average sleep onset, midsleep and sleep end, for each of the 19 students who used Fitbits, reveals a close correspondence between sleep diary and Fitbit data (average of all days of recording; Fig 1A). Correlations between the two data sets were uniformly high (e.g., *r* = .98 for MSF), and group means were not significantly different (Fig 1B and 1C). Estimates of nocturnal sleep duration (SD, after subtracting sleep-onset-latency from time-in-bed, and SD-WASO, further subtracting wake-after-sleep-onset) were also highly correlated (*r* = .69, *p* = .0011). Fitbits on average recorded 38 ± 34 mins less sleep per night than was reported in the diaries (6.87 ± .74 h v 7.51 ± .63 h, *t*_*18*_ = 6.315, *p* < .0001; Fig 1B and 1C). A difference in that direction is expected, given that accelerometer-based sleep trackers can more reliably measure and record sleep-onset-latency (SOL) and WASO compared to free recall.

Diary data also closely matched the questionnaire data. To conform with the MCTQ data, sleep timing and duration were calculated separately for work and free days. Midsleep time on free days calculated from the sleep diaries (MSF_diary_) correlated strongly with midsleep from the MCTQ (MSF_MCTQ_) in the Fitbit-user subgroup (Pearson *r* = .89, p < .0001; Fig 1D), the full 2020 group (*r* = .77, n = 78, p < .0001) and the archival group (*r* = .78, n = 416, p < .0001). Correlations between nocturnal sleep duration (SD) from the diary (SDF_diary_) and MCTQ (SDF_MCTQ_) data were lower but significant for the Fitbit subgroup (*r* = .55, *p* = .0181; Fig 1E), the full 2020 group (*r* = .42, *p* = .0001) and the archival group (*r* = .28, *p* < .0001). Similar correlations were evident for midsleep time and sleep duration on work days.

MSF_diary_ and MSF_MCTQ_ data were normally distributed in both the 2020 and archival groups, and distribution means and standard deviations did not differ between the two measures in either group. The distributions of sleep duration (nocturnal sleep onset to sleep end) from the diary and MCTQ data were also Gaussian but the means differed, with SDF_MCTQ_ > SDF_diary_ in both groups. Differences in sleep duration between measures and groups are explored further below.

The MEQ assesses time of day preferences, and correlates negatively with MSF_MCTQ_ in adult populations (higher MEQ scores associate with earlier MSF) [32]. Significant negative correlations between the MEQ and MSF were evident in the Fitbit subgroup (MSF_MCTQ_, *r* = -.58, *p* <. 01; MSF_diary_, *r* = -.35, *p* = .07), the full 2020 group (MSF_MCTQ_, *r* = -.77, *p* < .0001; MSF_diary_, *r* = -.63, *p* < .0001), and the archival group (MSF_MCTQ_ *r* = -.76, *p* < .0001; MSF_diary_ *r* = -.66, *p* < .0001).

The close match between the diary, Fitbit and questionnaire data provides confidence that sleep diaries submitted for university course credit yield estimates of sleep timing and duration comparable in validity to diary data from research volunteers.

### 3.2. Later nocturnal sleep timing in the social-distancing semester

**Fig 2:**
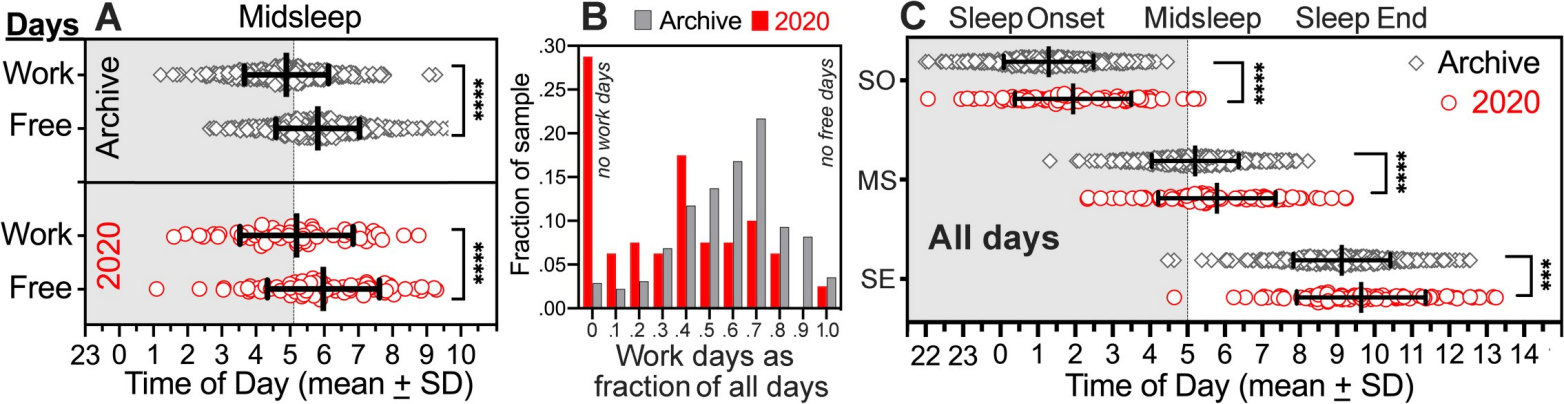
Sleep timing (diary data) on work, free and all days in the archive group (grey diamonds; N = 452) and the 2020 group (red circles; N = 80). [A]. Midsleep on work days (MSW) and on free days (MSFsc, sleep corrected). [B]. Frequency distributions of work days as a fraction of all days in the archive group (grey shaded bars) and the 2020 group (red open bars). The 0 bin denotes no work days, and the 1.0 bin denotes no free days. [C]. Sleep onset (SO), midsleep (MS, not sleep corrected) and sleep end (SE) averages for individual subjects across all days (bars denote group means ± standard deviation). ANOVA with Sidak multiple comparisons tests. ****p ≤ .0002, *** *p* = .001.

Midsleep times from sleep diaries were used as the primary phase marker for sleep timing, and were calculated separately for work and free days (Fig 2A). A mixed model 2-way ANOVA of midsleep revealed a significant main effect of group (2020 vs archive) (*F*_*1*,*529*_ = 5.245, *p* = .0224) and day type (work vs free) (*F*_*1*,*478*_ = 131.2, *p* < .0001), with no significant interaction (*F*_*1*,*478*_ = 0.184, *p* = .668). Within-groups, MSFsc (sleep corrected) was significantly later than MSW in both the 2020 group (05:53 ± 0:52 h vs 05:11 ± 01:40 h, *p* < .0001) and the archival group (05:38 ± 1:11 h vs 04:53 ± 01:14 h, *p* < .0001). Differences between work and free days were greater using uncorrected MSF, but the correction factor did not differ between groups (15±23 min vs 13±12min, *p* = .519). Between-groups, midsleep times were significantly later in the 2020 group on work days (17 min, *p* < .05) and free days (15 min, *p* < .05).

As a percentage of all recorded days, the 2020 group had 44% fewer work days than did the archival group (34 ± 28% vs 59 ± 21% of all days), and 28% of the 2020 group reported no work days at all (Fig 2B). True differences between groups are therefore potentially underestimated by comparing averages of work and free days separately. To adjust for group differences in the prevalence of work and free days, data for each subject were averaged across all days. By comparison with the archive group, sleep onset in the 2020 group on average was 35 min later (01:53 ± 01:38 h vs 01:18 ± 01:13 h, *t*_*530*_ = 3.714, *p* < .0001), midsleep was 31 minutes later (05:44 ± 01:39 h vs 05:13 ± 1:12 h, *t*_*530*_ = 3.298, *p* = .0002) and sleep end was 28 min later (09:35 ± 01:47 h vs 09:07 ± 01:22 h, *t*_*530*_ = 2.608, *p* = .0034) (Fig 2C).

### 3.3. Shorter nocturnal sleep duration in the social-distancing semester

**Fig 3:**
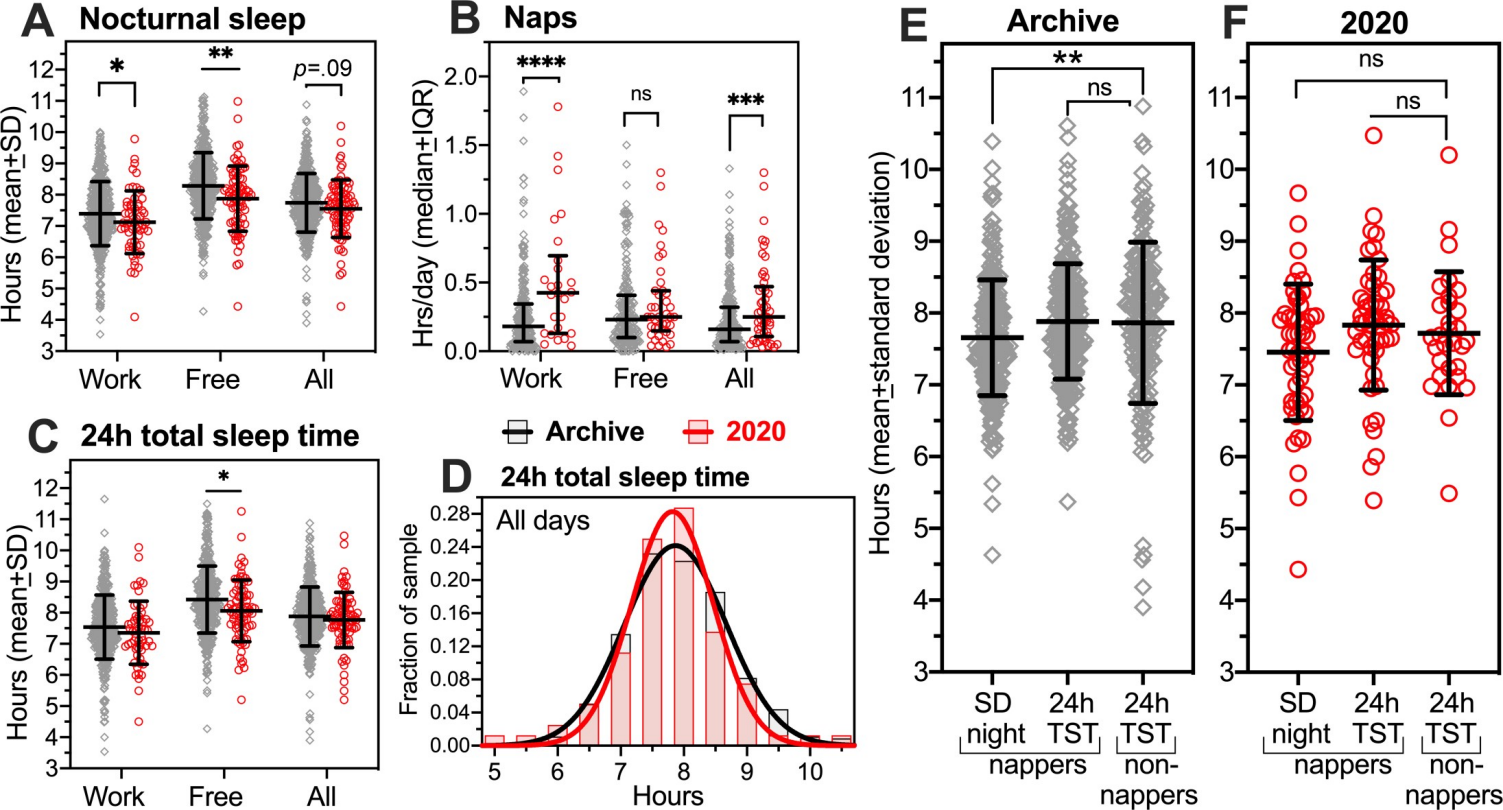
Nocturnal, daytime and 24h total sleep duration in archive (grey) and 2020 (red) groups. [A]. Group mean (± standard deviation) hours of sleep at night prior to work, free and all days (corrected for self-reported sleep latency and wake-after-sleep-onset). [B]. Group mean hours of daytime nap sleep. [C]. Group mean total daily sleep (sum of nocturnal sleep and daytime naps). [D]. Frequency distributions with gaussian curve fits of 24 h total daily sleep duration in the archive group (black curve) and the 2020 group (red curve). [E,F]. Nocturnal and 24h total sleep duration in nappers and non-nappers in the archive group (E) and the 2020 group (F). Panels A,C: (work x free days) ANOVA with Sidak multiple comparisons tests. Panel B: Mann-Whitney U test. Panels A (all days), B (all days), E,F: Unpaired t-tests. *p < .05, **p < .01, ****p < .0001.

Nocturnal sleep duration was calculated by subtracting reported SOL and WASO from time in bed with lights and media off. A mixed 2-way ANOVA revealed a significant main effect of group (*F*_*1*,*530*_ = 12.27, *p* = .0005) and day type (*F*_*1*,*478*_ = 105.1, *p* < .0001), with no significant interaction (*F*_*1*,*478*_ = 0.1735, *p* = .677) (Fig 3A). Within-groups, sleep duration was longer on free days compared to work days (*p* < .0001 for both groups). Between-groups, nocturnal sleep duration was shorter in the 2020 group by 16 min on work days (7.12 ± 1.0 h vs 7.39 ± 1.03h, *p* < .05) and 24 min on free days (7.88 ± 1.04h vs 8.29 ± 1.06h, *p* < .01). For all days combined, nocturnal sleep in the 2020 group was 12 min shorter than in the archive group, significant only at the level of a trend (7.55 ± .92h vs 7.75 ± .93 h, *t*_*530*_ = 1.66, *p* = .0973). The apparent discrepancy between the difference for all days and for work and free days separately is due to differences in group membership (not all subjects had both work and free days).

### 3.4. Daytime naps compensate for less nocturnal sleep

Shorter nocturnal sleep in the 2020 group was unexpected, but could reflect fewer constraints on daytime napping due to exclusively on-line, mostly asynchronous (recorded rather than live) classes and fewer work days. The percentage of students who reported napping was only slightly greater in the 2020 group compared to the archive (63% v 58%), and average nap duration was 9 min shorter (1.32 ± .78 h v 1.47 ± .9 h; n.s.), but the percentage of days on which naps occurred was more substantially different, with the 2020 group napping on 25 ± 18% of days, compared to 15 ± 12% in the archive (*Z* = 2.23, *p* = .025). Converting nap frequency and duration to average nap hours per day for all subjects revealed more daytime sleep in the 2020 cohort on work days (Mann-Whitney *U* = 1841, *p* = .0008) and all days combined (*U* = 5072, *p* = .0091) (Fig 3B). When naps were added to nocturnal sleep, there remained a significant main effect of group on total sleep time (*F*_*1*,*530*_ = 5.44, *p* = .02), but the group difference was significant only on free days (21 min less sleep in the 2020 group, *p* < .05), and not for all days combined (4.8 min less sleep in the 2020 group; 7.79 ± 0.88 h vs 7.87 ± 1.0 h) (Fig 3C and 3D). The 2020 group slept slightly less at night, compensated with slightly more daytime sleep, but did not obtain more sleep overall compared to the archival group. Across all days, 84% of the 2020 group averaged at least 7.0 h sleep per day, compared to 86% in the archive (*Z* = .435, *p* = .66).

The compensatory nature of naps was also made apparent by comparing sleep duration in nappers and non-nappers. In the archival group, nocturnal sleep in nappers was 14 min less than total daily sleep in non-nappers (*t*_*450*_ = 2.72, *p* = .0067); with naps added, total daily sleep duration in nappers and non-nappers differed by < 1 minute (Fig 3E). In the 2020 group (Fig 3F), nocturnal sleep in nappers was 13 min less than total daily sleep in non-nappers (*t*_*78*_ = 2.03, *p* = .21); with naps added, total daily sleep in nappers was 6 min greater than in non-nappers (*t*_*78*_ = 0.55, *p* = .58). Evidently, naps may compensate for nocturnal sleep but do not significantly increase total sleep time in nappers relative to non-nappers.

### 3.5. No difference in sleep duration in students reporting all free days

**Fig 4:**
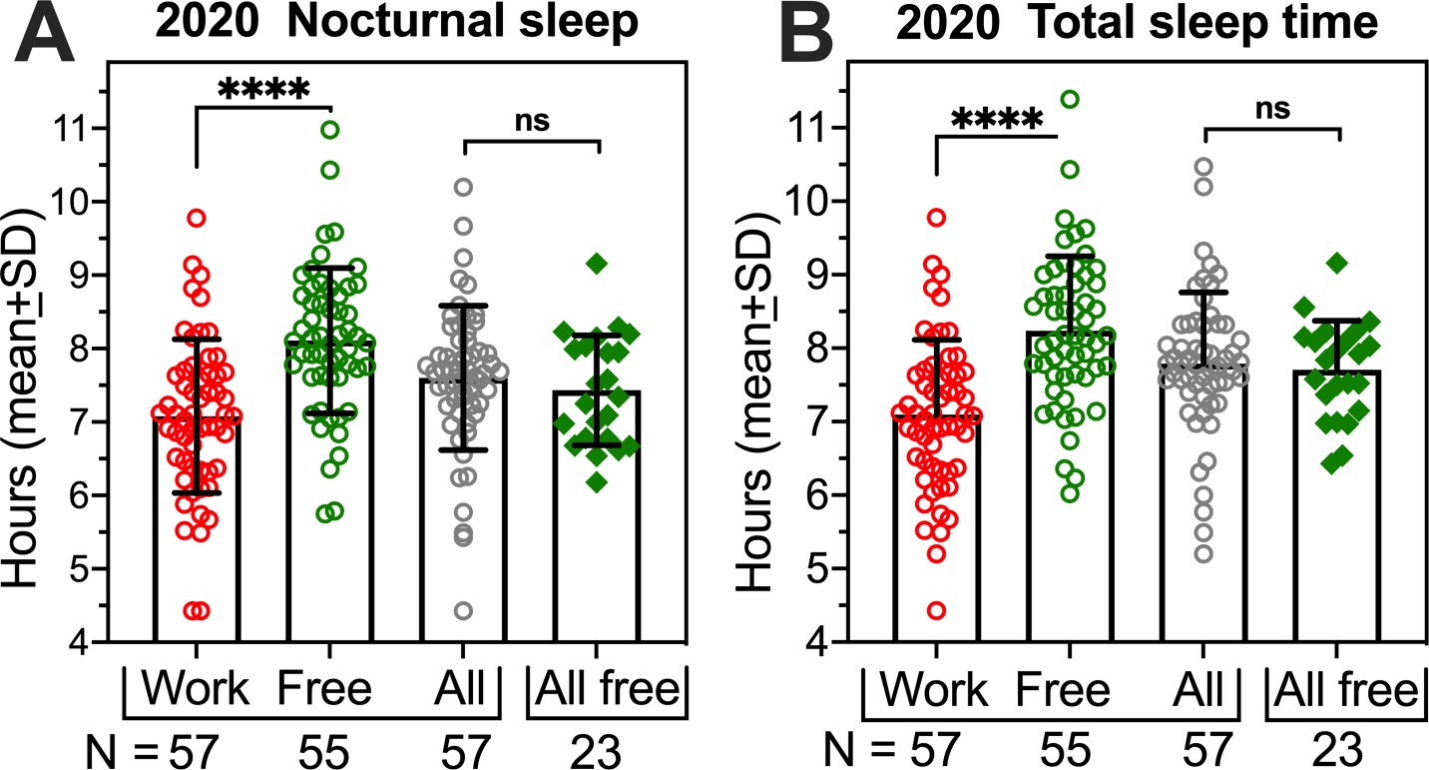
Nocturnal sleep duration (A) and total daily sleep duration (B) of participants in the 2020 group who had work days (N = 57) or only free days (N = 23). Paired t-tests for comparisons of work and free days, and unpaired t-tests for comparisons of average sleep across all days in those with work and free days (’all’) and those with only free days (’all free’). **** p < .0001.

The 2020 summer group was unique in the high percentage of students who reported no work days (28%), compared to the archival years (<2%). If work days (those requiring a scheduled wakeup for work, school or other obligation) normally contribute to a chronic sleep insufficiency, then students in 2020 reporting no work days might be expected to average more sleep across all days than students reporting a mix of work and free days. The results do not support this hypothesis. Students with work days did sleep less on nights prior to work, compared to nights prior to free days (paired *t*_*54*_ = 8.083, *p* < .0001; Fig 4A), but averaging across all days, these students obtained the same amount of sleep as students with only free days (a group difference of only 1.8 minutes; *t*_*78*_ = .746, *p* = .457). The same result was obtained when daytime naps were included; total sleep time (TST, nocturnal sleep plus naps the next day) was less for work days than free days (*t*_*54*_ = 8.719, *p <* .0001; Fig 4B), but across all days, TST did not differ between students with work days, and student with only free days (*t*_*78*_ = 0.701, *p* = .485)

### 3.6. Differences between MCTQ and diary may reflect the impact of social-distancing

**Fig 5:**
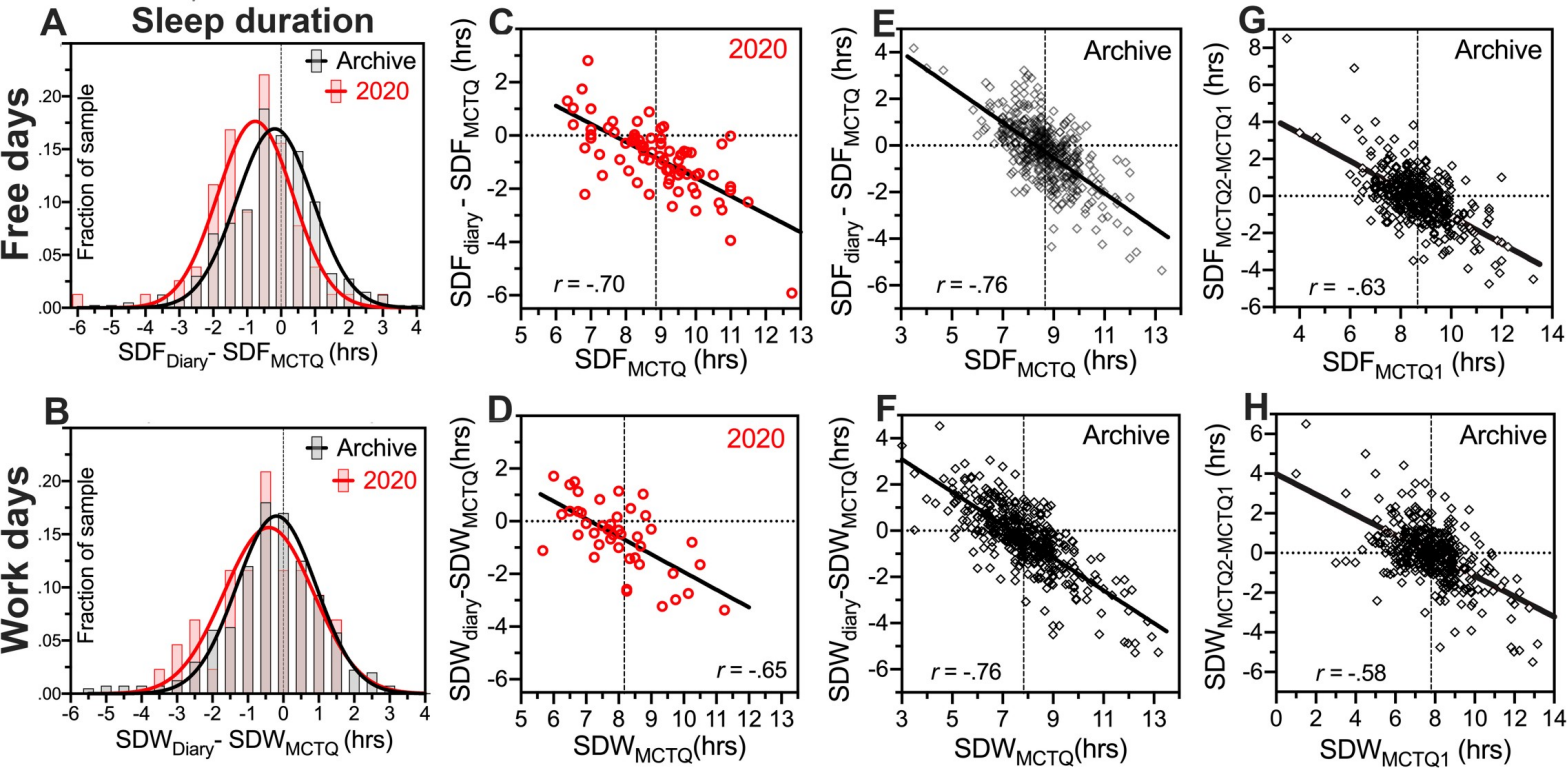
Differences in nocturnal sleep duration (SD) as measured by sleep diaries and Munich Chronotype Questionnaire (MSTQ) for free days (SDF_diary_, SDF_MCTQ_) and work days (SDW_diary_, SDW_MCTQ_). [A,B]. Frequency distributions and gaussian curve fits of signed differences between SDF_diary_ and SDF_MCTQ_ (A) and SDW_diary_ and SDW_MCTQ_ (B). [C-H]. Bivariate scatterplots, with Pearson r correlation coefficients and best fitting linear regression lines. [C,D]. Relationship between SD_MCTQ_ and the signed difference between SD_diary_ and SD_MCTQ_ for work days (C; N = 43 pairs) and free days (D; N = 78) in the 2020 group. [E,F]. Relationship between SD_MCTQ_ and the signed difference between SD_diary_ and SD_MCTQ_ for work (E; N = 400) and free days (F; N = 398) in the archive group. [G,H]. Relationship between SD measured by MCTQ early in the semester (SD_MCTQ1_) with the difference between SD_MCTQ1_ and SD measured by MCTQ a second time, late in the semester (SD_MCTQ2_) for free days (G; N = 402) and work days (H; N = 388) in the archive group. r denotes Pearson correlation coefficient. Regression lines fit by linear least squares.

In their written reports, 26 of 80 students in the 2020 group made explicit statements that the MCTQ data, which they input early in the summer semester, corresponded better to sleep habits during the previous semester, prior to social-distancing measures (e.g., *"The MCTQ was administered reasonably early on into my remote learning and remote work situation*, *and so the lifestyle changes that resulted from COVID-19 were less prominent at the time*. *The questions were therefore answered in a way that is more reflective of my pre-COVID-19 sleep habits*. *If I were commuting to work and school as normal and were still active with all of my volunteer commitments*, *my sleep log would likely be more similar to the MCTQ results"*). If this was generally true for many if not all students in 2020, then differences between MCTQ and sleep diary data may reflect in part the impact of social-distancing measures. For free days, the duration of the sleep period (sleep onset to sleep end) calculated from the MCTQ (SDF_MCTQ_) exceeded the diary averages (SDF_diary_) by 18 ± 64 min in the archival group (N = 398 with free days and both MCTQ and diary data), and by 52 ± 65 min in the 2020 group (N = 78), a significantly larger difference (between-groups *t*_*473*_ = 3.341, *p* = .0009, Fig 5A). For work days, SDW_MCTQ_ exceeded SDW_diary_ by 19 ± 86 min in the archival group and 36 ± 78 min in the 2020 group (between-groups *t*_*441*_ = 2.952, *p* < .0033, Fig 5B). Self-reported sleep duration is overestimated in questionnaires compared to diaries [34,35]. The archival group data suggests that in this student demographic, the overestimate is about 18–19 minutes. In the 2020 group, the discrepancy was 2.8 times larger on free days (34 minutes) and 1.9 times larger on work days (17 minutes). The differences may be larger in the 2020 group not due to greater misperception of sleep duration in the MCTQ, but because students were in fact sleeping less at night during the social-distancing semester compared to previous semesters. This interpretation is consistent with student statements that the MCTQ data fit better with sleep habits prior to the transition to remote classes.

### 3.7. Differences between MCTQ and diary may also reflect regression to the mean

During a COVID-19 ’stay-at-home’ week, average time-in-bed (TIB) in a class of university students increased by ~26 minutes compared to a baseline week prior to the ’stay-at-home’ orders [25]. Notably, the change in TIB correlated negatively with baseline TIB (*r* = -.51 for work days, *r* = -.70 for free days); students who recorded shorter TIB at baseline recorded larger increases in TIB during ’stay-at-home’. If in our sample the MCTQ represents ’pre-social-distancing’ sleep habits, then we might also see a negative correlation between sleep duration at ’baseline’ (i.e., SD_MCTQ_) and the amount by which sleep changed from baseline during the social-distancing semester (i.e., SD_diary_-SD_MCTQ_). Consistent with this hypothesis, a negative correlation of very similar magnitude to Wright et al. [25] was evident for work days (*r* = -.70, *p* < .0001; Fig 5C) and free days (*r* = -.65, *p* < .0001; Fig 5D). Students who slept less than the group average according to the MCTQ (the ’pre-COVID19 baseline’), tended to report longer sleep in their diary compared to their own MCTQ.

While this finding would seem to bolster the conclusion that ’stay-at-home’ and social-distancing measures reveal a subset of students who are sleep restricted at baseline and who benefit from having more time available for sleep, it is only half the story. The negative correlations include many students who slept more than the group average at time 1 (diary 1 in [25], MCTQ in our study) and then reported shorter sleep at time 2 (diary 2 in [25], diary in our study). A negative correlation between SD_MCTQ_ and SD_diary_-SD_MCTQ_ was also evident in our archival sample, for free days (*r* = -.76, *p* < .0001; Fig 5E) and work days (*r* = -.76, not a typo, p < .0001; Fig 5F). Students in the archival group were required to complete the MCTQ and MEQ twice, once at the beginning of the semester and once at the end, to assess the potential effect of keeping a sleep diary on questionnaire responses. Although there was no significant mean difference between the two time points for any variable (e.g., sleep duration, midsleep time or MEQ score), the standard deviations of the differences were of course not zero. Again, the change in sleep duration from time 1 to time 2 (SD_MCTQ2-MCTQ1_) correlated negatively with sleep duration at time 1 (SD_MCTQ1_), for work days (Fig 5G) and free days (Fig 5H). The association was also evident for midsleep time on work and free days, and for MEQ scores. Whether or not group mean TIB or sleep duration change when more time is available for sleep, the association between the value at time 1 and the change from time 1 to time 2, at the group level likely reflects statistical regression to the mean with repeated sampling.

### 3.8. Reduced social jetlag burden in the social-distancing semester

**Fig 6:**
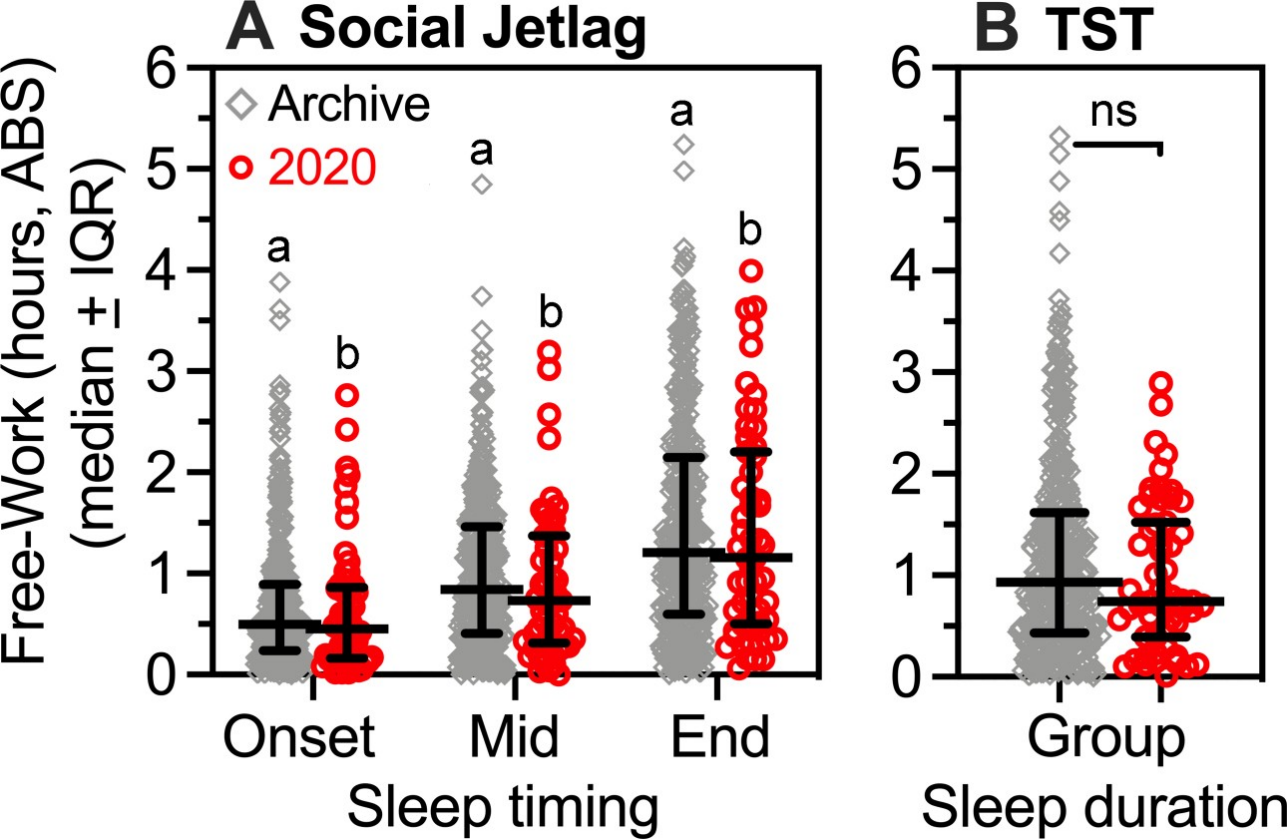
Differences between work and free days in sleep timing (social jetlag) and sleep duration. **[**A]. Absolute value of differences between work and free days in sleep onset, midsleep, and sleep end in the archive (grey diamonds) and 2020 groups (red circles). Variables within groups that share a letter (a or b) are significantly different from each other by Friedman test (archive group N = 422, χ^2^_2_ = 256.7, p < .0001; 2020 group N = 55, χ^2^_2_ = 32.2, p < .0001). [B]. Absolute value of differences between work and free days in total sleep time. In panels A and B, differences between groups are not significant (Mann-Whitney U tests).

Social jetlag refers to a misalignment between social time and biological (circadian) time, which can be inferred from differences in sleep timing on free days compared to work days. As reported above, MSF was significantly later than MSW in both the archive and 2020 groups. To further explore social jetlag, average sleep onset, midsleep and sleep end times for work days were subtracted from averages for free days for each student, and differences converted to absolute values. In both the 2020 and archival groups, differences between work and free days were smallest for sleep onset (~40 min) and largest for sleep end (~85 min), with mean differences ~5 min smaller (n.s.) in the 2020 group (Fig 6A; Friedman one-way ANOVA within groups, with Dunn’s multiple comparisons test). Differences between groups were not significant for onset (Mann Whitney *U* = 10979, *p* = .516), midsleep (*U* = 10384, *p* = .204) or sleep end (*U* = 11282, *p* = .737).

As reported above, the archival and 2020 groups averaged significantly more sleep on free days than on work days, but the magnitude of the differences expressed as absolute values did not differ between groups (*U* = 10823, *p* = .407; Fig 6B).

The cumulative biobehavioral impact of social jetlag presumably depends on both the magnitude of misalignment and the prevalence of misaligned days. Students in the 2020 cohort reported fewer work days (575 of 1,592 days total, compared to 8,909 of 14,883 days in the archive), so the collective burden of social jetlag in 2020 summer semester was substantially lower than in previous summer semesters (~44% lower if comparing ratios of work days to all days), even if the groups did not differ in the magnitude of the differences between work and free days.

### 3.9. Less variable sleep timing and duration in the social-distancing semester

**Fig 7:**
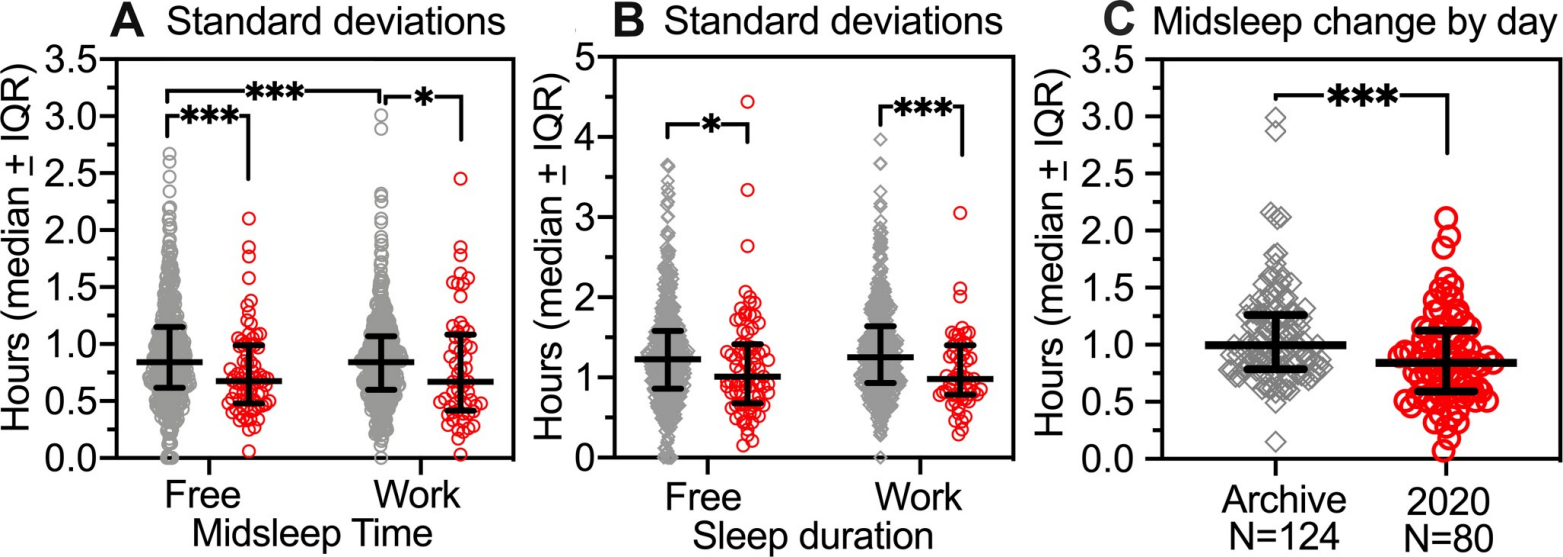
Within-subject variability (average standard deviations) of sleep timing and duration. [A]. Individual standard deviations of midsleep time in the 2020 group (red shading; N = 57 work days, N = 78 free days) and archive group (grey diamonds; N = 439 work, N = 431 free) for subjects with more than one work or free day. [B]. Individual standard deviations of sleep duration. [C]. Average daily change (absolute values) of midsleep time for individual subjects in the 2020 group and in a subset of the archive group. Between-group contrasts by Mann-Whitney U test. Within-group contrasts by Wilcoxon matched pairs signed rank test. *p < .05, ***p < .001.

Transitions between work and free days are one major source of variability in sleep timing and duration. Work and free days considered separately also exhibit significant variability, evident in non-zero standard deviations of midsleep times and sleep durations within subjects. Midsleep time variability was significantly lower in the 2020 group compared to the archival group on free days (Mann Whitney *U* = 12629, *p* < .0005) and work days (*U* = 10544, *p* < .05) (Fig 7A). Within groups, midsleep variability was lower on work days than free days in the archive group (Wilcoxon matched-pairs signed rank test, *p* = .0002), but not the 2020 group (*p =* .873). Individual standard deviations of sleep duration also exhibited a group difference, with variability significantly lower in the 2020 group on work days (*U =* 8195, *p* = .0001) and free days (*U =* .13730, *p* = .0243; Fig 7B). Variability of sleep duration on work and free days did not differ within groups.

Variability of sleep timing around the mean for an individual does not provide information on how large the variation is from day to day. To quantify daily variability, the absolute value of the average change of midsleep between successive days was calculated across all days in the 2020 group and in a subset of the archive (N = 124) for which these data were available. Individuals in the 2020 group averaged significantly smaller daily changes in midsleep time (Mdn = 0.84 h) than did individuals in the archival subsample (Mdn = 0.99 h, *U* = 3501, *p* = .0004; Fig 7C).

### 3.10. Lower nocturnal sleep efficiency in the social-distancing semester

Sleep efficiency is quantified as the ratio of time asleep to time in bed intended for sleep, and predicts subjective sleep quality [36,37]. To quantify sleep efficiency from sleep diaries, subjects must provide estimates of SOL and WASO, which are then subtracted from time in bed with lights and media off. Students were instructed that sleep latency is difficult to estimate accurately, and that brief awakenings at night are typically not recalled, but that if students do remember being awake prior to sleep onset or during the night, they should record an estimated duration. These instructions were intended to capture potential issues such as sleep onset insomnia or other sleep disruptions that can be expected to occur in some percentage of students in any year, and that might be exacerbated by social-distancing measures.

**Fig 8:**
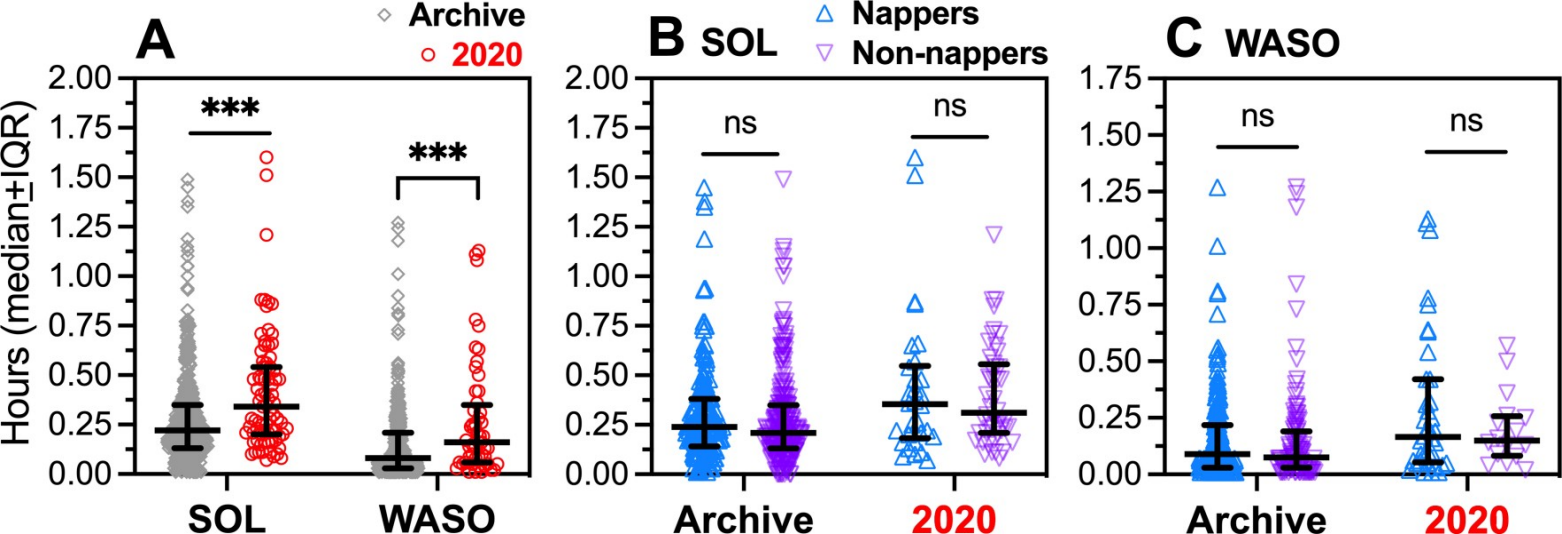
Sleep onset latency (SOL) and wake-after-sleep-onset (WASO) for the 2020 group (red circles, N = 80) and archive group (grey diamonds, N = 452), averaged across all days (work and free). [A]. All subjects. [B]. SOL in nappers (archive group N = 183, 2020 group N = 50) and non-nappers. [C]. WASO in nappers and non-nappers. Between-group contrasts by Mann-Whitney U test. *p < .05, **p < .01, ***p < .0005, ****p < .0001.

Despite the limitations of memory, the self-reports of SOL and WASO appear to yield meaningful data. In the 2020 Fitbit-user group, average self-reported WASO was significantly lower than WASO detected by Fitbits (median = .22 h v .90 h, *W* = 62, *p* = .011), but the two measures nonetheless correlated significantly (Spearman *r* = +.79, p = .0034). In the full 2020 group, across all days, both SOL and WASO were significantly elevated compared to the archival group (Fig 8A; SOL, Mdns = 0.34 h v 0.22 h, *U* = 12193, *p* < .0001; WASO, Mdns = 0.16 h v 0.08 h, *U* = 5834, *p* = .0005). As a result, sleep efficiency was significantly lower in the 2020 group (Mdns = 0.945 v 0.960, *U* = 13183, *p* < .0001).

Sleep efficiency may be negatively impacted by daytime napping. A number of students noted in their written reports that they normally resist napping to avoid sleep problems at night. Nonetheless, daytime nap hours were elevated in the 2020 group compared to the archival group. To evaluate the role of daytime naps in group differences in SOL and WASO, the 2020 and archival groups were divided into napping and non-napping subgroups. SOL was higher in the 2020 group compared to the archival group in both nappers (Fig 8B; 2020 N = 50, archive N = 265; Mdn = 0.36 h v 0.24 h, *U* = 2144, *p* = .027) and non-nappers (2020 N = 30, archive N = 187; Mdn = 0.31 h v 0.21 h, *U* = 4055, *p* = .0003). WASO was also higher in the 2020 group compared to the archival group in both nappers (Fig 8C; Mdn = 0.17 h v 0.09 h, *U* = 2697, *p* = .0069) and non-nappers (Mdn = 0.15 h v 0.08 h, *U* = 580.5, *p* = .0218). Averaged across all days, SOL was slightly but not significantly elevated in nappers compared to non-nappers in both the 2020 group (*U* = 679.5, *p* > .1) and archival group (*U* = 21350, *p* = .189) (Fig 8B). WASO was similarly elevated slightly but not significantly in nappers compared to non-nappers, in both the 2020 group (*U* = 271.5, *p* > .1) and archival group (*U* = 11116, *p* > .1) (Fig 8C). The results therefore do not provide clear support for a hypothesis that increased daytime napping accounts for the reduced sleep efficiency in the 2020 group.

### 3.11. Low outdoor light exposure in the social distancing semester

**Fig 9:**
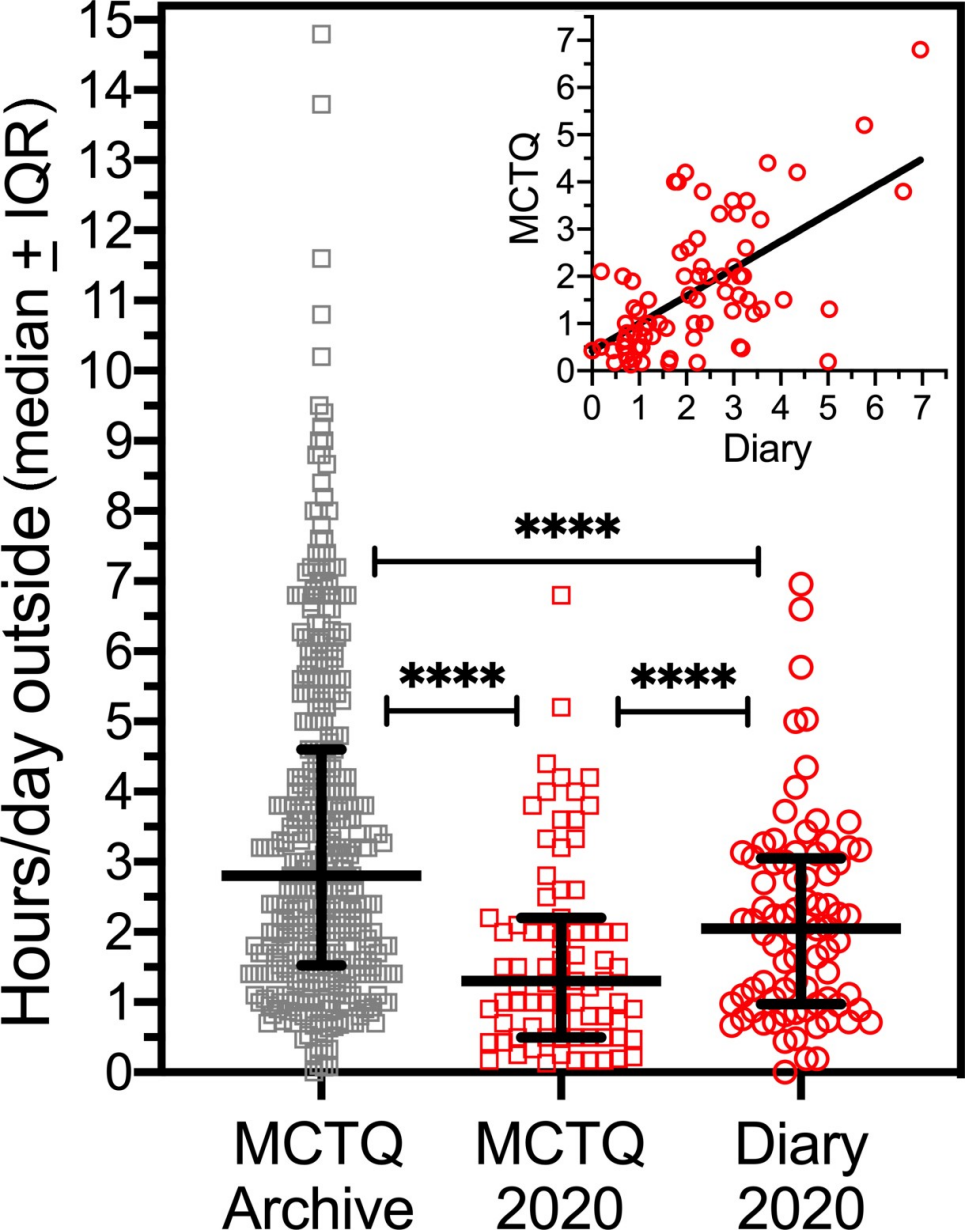
Hours outside during daylight in the archive group (grey symbols) and 2020 group (red symbols), from the MCTQ (squares) and sleep diaries (circles). Inset panel: Bivariate plot of diary and MCTQ data in the 2020 group (Spearman r = .548, *p* < .0001). ****p < .0001.

Light, especially in the morning, is the primary environmental time cue for entraining the human circadian sleep-wake cycle to local time [27]. Reduced outdoor light exposure due to social-distancing measures may explain delayed sleep timing in the 2020 group. Consistent with this hypothesis, estimates of light exposure reported on the MCTQ were significantly lower in the 2020 group compared to the archive, on work days (median difference = 1.0 h, *U* = 5442, *p* < .0001) and free days (median difference = 1.67 h, *U* = 8949, *p* < .0001; Fig 9). The 2020 group was asked to record in their diaries the time of first outdoor light exposure and total outdoor light exposure each day. Across all days, the median first outdoor light exposure was 12:42 pm (mean = 12:30pm), with a median daily outdoor duration of 1.30 h (mean = 1.68 ± 1.38 h). Students reported going outdoors on 78 ± 28% of recorded days (range 0 to 100%). Two students reported no outdoor sunlight exposure for the entire duration of data collection (14 and 18 days, respectively). Information on time outdoors was not recorded in the archival sleep diaries, but based on MCTQ estimates, daylight exposure in the 2020 cohort was likely reduced by 50% or more, and likely occurred later in the day, given the fewer work days and asynchronous, on-line classes. This interpretation assumes that the MCTQ estimate of time outside in the 2020 group represents behavior during social-distancing, rather than prior to social-distancing, as suggested by the MCTQ sleep duration data. Both may be partially true, depending on the subjective salience of recent changes in time outdoors (decreased by 50%?) and sleep patterns (~30 min later bedtime).

### 3.12. Age and sex differences in sleep timing and duration

Age and sex differences in sleep timing and duration have been well documented, and were evident in our samples. MSF advances with age [27,38], and despite the restricted age range in our subject pool, a negative correlation between MSF_diary_ and age was evident in both the archival group (Pearson *r* = -.217, *p* < .0001) and the 2020 group (*r* = -.304, *p* = .0068). Sleep duration is also negatively correlated with age [7,8], and this was evident in the diary data for total sleep time (all days) in both the archival group (*r* = -.138, *p* = .0037) and the 2020 group (*r* = -.161, *p* =. 153). MSF is advanced in females compared to males, up to the age of menopause [10], and this sex difference was evident in MSF_diary_ in both the archival group (5.71 vs 5.98, *t*_*429*_ = 2.155, *p* = .016, 1-tailed) and the 2020 group (5.52 ± 1.70 v 6.28 ± 1.39, *t*_*78*_ = 1.868, *p* = .033, 1-tailed). No sex differences were evident in nocturnal or total daily sleep duration. The degree of conformity between these data and known age and sex differences in sleep timing and duration underscores the validity of the student sleep diary as a measurement tool for sleep in this population.

The 2020 and archival groups did not differ in average age or sex ratio. Given the narrow age distribution (90% between ages 19–26 in both groups), the small percentage of males (28% and 29%, respectively), and the lack of hypotheses concerning interactions with social-distancing measures, age and sex were not considered further.

### 3.13. Subjective impact of social-distancing on sleep varies with chronotype

For their written reports, students in the 2020 group were asked to evaluate the impact of the COVID-19 social-distancing measures on their sleep. The instructions did not include a checklist of factors to consider, and therefore the content of the discussion presumably reflects the factors that were most important or salient to their individual experience. Most of the students assessed the impact as big (65%) or moderate (28%), with only two noting little or no impact. A majority of students (74%) stated that bedtime and wakeup time were later than usual; actual midsleep time (all days) in this subgroup was significantly later compared to students who did not make this explicit statement (mean difference = 56 min, *t*_*78*_ = 2.359, *p* = .0208). Some students noted that they liked the shift to later sleep times (e.g., *"the COVID-19 pandemic has been beneficial in allowing me to wake up at my preferred time"; "elimination of the commute has allowed me to sleep in and I am now able to wake up for classes at a time much more preferable to my personal needs*, *and the commute time can now be spent sleeping*, *which has eliminated my social jetlag*"). Some others expressed dissatisfaction with later sleep times (e.g., *"I definitely sleep much later than I did regularly before COVID-19*, *and my sleep consistency is also much worse*. . . *COVID-19 has acted more as an enabler to bad sleeping habits than anything else"; "the day is almost over by the time I get up*"). Sleep timing was described by equal numbers of students as either more regular (23.1%) or less regular, even "*chaotic*" (24.4%).

A substantial proportion of students (35%) stated that they were sleeping more than usual, although total sleep time reported by these students did not differ from the other students (mean difference = 7.8 min, *t*_*78*_ = 0.62, *p* = .53). A small number of students (10.3%) felt they were sleeping less than usual. Average sleep duration across all days in this subgroup was 25.8 min shorter than the other students (*t*_*78*_ = 1.30, *p* = .196). Total sleep time was also slightly although not significantly less in this subgroup compared to those who felt they were sleeping longer (mean difference = -28.2 min, *t*_*33*_ = 1.38, *p* = .176).

Nearly a quarter (or ’only a quarter’) of the students (24.4%) reported feeling better rested (e.g., "*As a demonstrable night owl*, *I’ve found the alleviation of morning work responsibilities to have done great things for my subjective experience of sleep quality*. *I undoubtedly feel more rested and far less tired than I did this time last year despite having roughly the same amount of weekly work"; "my sleep quality has drastically improved in the past few months*. *I am able to sleep and wake up at consistent times*, *as well as having longer sleep durations*, *without the use of an alarm compared to pre-COVID-19"; "Throughout this period*, *I have felt consistently rested"; " I do tend to feel well rested when I wake up on most days*. . .*this is most likely because for the period I kept a diary*, *I was able to sleep in as long as I needed*"). Despite feeling better rested, sleep duration in this subgroup was not significantly different from the other students (mean difference = 17.4 min, *t*_*78*_ = 1.26 *p* = .208).

A smaller number of students reported feeling less rested (14.1%) (e.g., "*Although I have more hours of sleep now as compared to prior covid-19*, *I feel that my quality of sleep has worsened*. *Even if I slept for 8–9 hours*, *I still feel tired"; "although all of my data was recorded on free days*, *I did not feel particularly well rested or energetic throughout the 14 day period*. *My sleep data shows that I slept and woke at inconsistent times*, *and even slept for inconsistent durations*. . . . . *despite all of the days being free days*, *I actually felt better when I was sleeping more consistently during normal in-person school semesters*"). Again sleep duration in these students did not differ from the others (mean difference = 9.6 min, *t*_*78*_ = 0.56, *p* = .57).

Difficulty falling asleep was reported by 10.3% of students, with all but one of these individuals noting a history with this problem. Self-reported SOL in these students was 45 ± 36 min, compared to 21 ± 12 min in students who did not note a sleep onset problem (*t*_*76*_ = 3.914, *p* = .0002).

**Fig 10:**
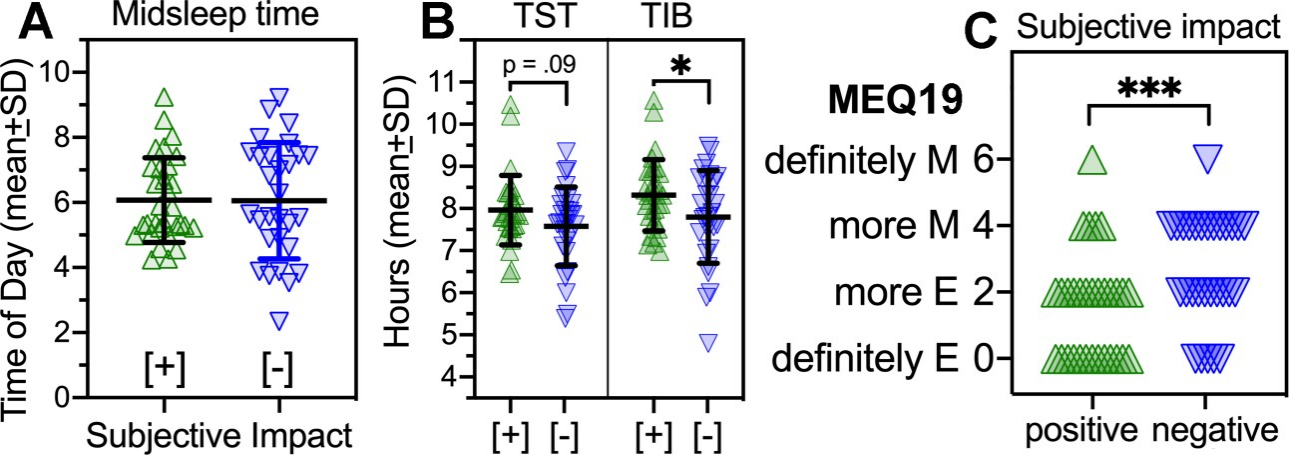
Midsleep time, sleep duration and morning-evening preference in relation to an inferred net positive (+) or negative (-) response to sleep changes during the social-distancing semester in the 2020 group. **[**A]. Midsleep time, across all days. [B]. Left panel: Total daily sleep duration, across all days. Right panel: Nocturnal time in bed (TIB). Independent samples t-tests, *p < .05 [C]. Self-identified evening-preference, from question 19 on the Morning-Evening Questionnaire (MEQ). Mann-Whitney U test, ***p < .001. Green triangles denote positive responders (N = 31). Purple inverted triangles denote negative responders (N = 29).

The subjective impact of social-distancing on sleep was clearly heterogenous within the class, as illustrated by the representative comments. From these descriptions, impact was rated as either net positive or negative (see Methods for criteria). Of the 80 students, a net positive impact was inferred in 31 cases, a net negative impact in 29 cases, and neutral (or unassigned) impact in 20 cases. The net positive and negative groups did not differ in sleep timing (midsleep across all days, mean difference = 1.2 min), but the net positive group averaged more time in bed (*t*_*58*_ = 2.021, *p* = .048) and a trend for more sleep (*t*_*58*_ = 1.69, *p* = .0946) (Fig 10A and 10B). The groups did not differ in reported time of first outdoor light exposure or amount of time spent outdoors.

Although not associated with objective sleep timing (midsleep from diary data), subjective impact was associated with morning-evening preference. Based on the response to question 19 of the MEQ (*’do you see yourself as a definitely morning type*, *more morning type*, *more evening type*, *or definitely evening type’*), 48% of the students in the negative impact group self-identified as a morning chronotype (more or definitely morning), compared to 16% in the positive impact group. By contrast, 42% of the students in the positive impact group identified as a definitely evening type, compared to 17% in the negative impact group (Fig 10C). Also, the median MEQ score was significantly lower (more evening) in the positive impact group (*U* = 277, *p* = .0066).

## 4. Discussion

Social-distancing orders during a pandemic can be expected to affect sleep, by changing daily work and school schedules, and altering behaviors that impact sleep timing and duration. Given that sleep is important for immune function and mental health [39,40], an important research agenda is to determine whether any effects of social-distancing on sleep are a harm or a benefit. Young adults are thought to regularly carry significant sleep debt related in part to misalignment between endogenous circadian clock time and social time [9,10]. We would therefore expect average sleep duration to increase if more time is made available for sleep during a pandemic, due to less time spent commuting, at work, or at social events. Changes in sleep timing, sleep efficiency, and daytime napping might be expected as well, although it is not certain that the net effect would be beneficial in all individuals.

Our analyses of student sleep diaries and reports collected during the 2020 summer semester at Simon Fraser University are in partial agreement with other recent findings. The most commonly reported effect of social-distancing has been a shift to later bedtimes, e.g., [12–25]. Compared to students in previous summers, students in our 2020 summer group showed a significant delay in timing of the daily sleep-wake cycle. The magnitude of the shift averaged across all days (work and free) was ~30 minutes, which is very similar to other reports based on MCTQ data (MSF later by 34 min, MSW later by 22 min) [14] and sleep diary data (midsleep later by ~32 min, assuming 5 workdays and 2 weekend days) [25]. Our 2020 group also showed less variability in sleep timing, less cumulative social jetlag due to fewer work days, and reduced sleep efficiency due to increased WASO. These differences fit a coherent narrative. Fewer work days combined with on-line, asynchronous classes should reduce or eliminate morning commutes, thereby decreasing or delaying exposure to outdoor light, especially early in the day. Removal of constraints on sleeping late permits later bedtimes, extending the duration of exposure to artificial light at night. According to principles of circadian clock entrainment, decreased morning daylight combined with increased evening artificial light will favor a delay shift in the phase of entrainment, and therefore later bedtimes and wakeup times [27]. A reduced amplitude of the daily light-dark cycle (weaker zeitgeber) should also amplify the natural tendency of young adults toward an evening chronotype [41,42]. Reduced constraints on daytime napping would also favor later bedtimes, more time awake in bed, and lower sleep efficiency. With fewer work days, by definition there would be less social jetlag, contributing to less variability in sleep time from day to day.

Contrary to prediction, we did not find evidence of increased sleep duration. Our sample of 80 students from the 2020 summer semester reported significantly fewer work days, and less cumulative social jetlag, compared to the age- and sex-matched archival sample of 452 students from previous summer semesters, yet they slept slightly less at night. This was offset by a trend for more daytime sleep, but average 24 h sleep time in the 2020 group was still a few minutes less than in the archival group. Similar findings of reduced nocturnal sleep, with or without increased daytime napping, have been reported in some survey studies of adults in the general population, e.g., [15–17,19–21]. Notably, in our 2020 group, 23 students who reported no work days averaged the same amount of sleep as 55 students reporting a mix of work days (prior to which they slept less) and free days (prior to which they slept more). In both the archival and 2020 groups, nappers slept less at night than non-nappers, but 24 h sleep time did not differ.

One interpretation of these data is that sleep in this particular young adult cohort is in homeostatic balance. Within a circadian cycle, naps appear to compensate for nocturnal sleep deficiencies, while across the week, free days appear to compensate for any sleep deficiencies on work days. Students during the 2020 summer semester had fewer work days, and fewer or no commutes to work and school, yet as a group they did not sleep more over periods of sleep tracking ranging from 14 to 56 days. This does not mean that students do not sustain periods of sleep restriction. What it does suggest is that at the group level, the average amount of sleep reported by these students over a period of 2 or more weeks, reflects a homeostatic system in balance; the data provide no evidence of a chronic sleep deficit that would be relieved by more time available for sleep. Young adults might be able to sleep more than they normally do (e.g., during forced bedrest in long nights in a laboratory), but when provided the opportunity in the home environment, this particular cohort, on average, did not.

This interpretation is in apparent disagreement with a broad consensus that modern life is forcing an unhealthy sleep diet on many people, especially youth. Other interpretations therefore bear careful consideration. One possibility is that sleep duration in the 2020 group failed to increase over previous years because with more flexibility and time available (less summer employment and no time spent commuting), students procrastinated on school work and then worked late to complete assignments. Even if freed from a social clock, sleep is nonetheless constrained by circadian clock alerting, which limits sleep in the biological day, e.g., [11,43]. This surely describes the situation for at least some students. Sleep may not have increased in other individuals due to general stress associated with health concerns, social isolation or other responses to the pandemic.

A counter to this argument is that if students in the 2020 group did self-restrict and carry a significant sleep debt, or experience sleep disruptions due to stress, we would expect to find evidence of this in their written reports. Only a few students reported that they did not feel well-rested during the self-study, and this may well have been due to other factors that affect daytime alertness and mood, such as reduced light exposure, exercise, or social interactions. Notably, these students did not average less sleep than the others. Very few students made a reference to stress; ennui appeared to be a more common state. The data provide no evidence that during COVID-19 social-distancing, many students wanted to sleep more, but were unable or could not find the time.

Our findings on sleep duration differ from those reported in at least two other studies of sleep during social-distancing in young adults. Participants in one study completed an on-line version of the MCTQ modified to include questions about habitual bedtimes and wakeup times both before and during social-restriction [14]. This memory-based, quasi-longitudinal approach revealed a net increase of ~15 min/day of sleep during social-restriction (less during free days but more during work days). Participants in the other study were students in a university course who maintained a sleep diary for one week prior to ’stay-at-home’ orders in late January 2020, and again 3 months later when ’stay-at-home’ orders were in effect [25]. Time in bed increased by ~26 min/night during the ’stay-at-home’ week. Whether sleep duration increased commensurately with TIB in these two studies is unknown, but it might not have, if sleep was less efficient, as appeared to be the case in our 2020 group when compared to previous years. We also note that the tendency for presumed short sleepers (short time in bed) to increase TIB during social restriction, as reported in both of these studies, is not in itself evidence of sleep deficit; long sleepers changed TIB in the opposite direction. The negative correlation between a first measure of sleep duration (or midsleep time, or MEQ) and the difference between a second and first measure, is a characteristic of repeated sampling and measurement error (regression to the mean). In other respects, the results of these two studies align well with our study (later bedtimes, reduced variability of sleep timing, reduced social jetlag burden).

Strengths of our study include the use of multiple instruments to characterize sleep and chronotype, the availability of written reports to help interpret the 2020 sleep data, the relatively large sample of sleep diaries and long periods of sleep tracking, the age- and sex-matching of the comparison groups, and the control for time of year. A limitation of the study is that given its exploratory nature, a large number of analyses were conducted, increasing the risk of Type 1 error. Familywise error rate was controlled using Dunn’s or Sidak’s post hoc tests. A second limitation of the study is the lack of data from individual students prior to social distancing that would allow us to report quantitatively on change within-subjects, rather than infer change (or lack of change) based on cross-sectional comparison with prior years or within-subject MCTQ (with some confirmation from analysis of the students’ written reports). Interpretation of the sleep duration data would also have benefited from more quantitative assessment of sleep quality (e.g., using the PSQI) and daytime functioning (e.g., sleepiness scales or regular tests of psychomotor vigilance). Generalizations from our study are limited by the narrow demographic of the study sample; mostly early 20’s in age, mostly female (70%), and self-selected (they chose to take this course on sleep, a course that has always been scheduled in the afternoon rather than in the morning). A limitation of the published literature to date is the paucity of objective data from validated sleep trackers, e.g., Actiwatches or Fitbits [22] or ambulatory polysomnography (PSG). Retrospective questionnaires generally yield overestimates of sleep duration relative to daily diaries, which yield overestimates relative to actimetry and PSG. The COVID-19 pandemic may precipitate a permanent movement toward increased work and school from home, which may increase time available for sleep, and how this might affect sleep behavior, efficiency and architecture will be important to evaluate further.

Camping outdoors, in a group setting, without access to artificial light, has been shown to rapidly advance the phase of circadian clock entrainment sufficient to largely eliminate social jetlag and individual differences in circadian timing [41,42]. COVID-19 social-distancing measures in many respects represent the converse experiment, in which natural circadian time cues (primarily sunlight exposure) are degraded rather than enhanced. The changes observed in sleep timing during social distancing add to the evidence that the built environment in modern industrialized societies promotes a delayed phase of circadian clock entrainment, which under normal circumstances in many people is misaligned with the social clock. The impact of these changes, however, may be positive for some but not for others. Individuals complying with mandates to social-distance or quarantine may therefore benefit from public health advice on sleep hygiene and effects of natural and artificial light exposure on sleep timing to maximize the benefits of good sleep for immune function and mental health.

## Supporting information

S1 File (XLSX)
